# Self‐limited familial focal epilepsy caused by *ANK2* variants: A potentially under‐recognized condition

**DOI:** 10.1002/epi4.70003

**Published:** 2025-02-17

**Authors:** Po‐Hsi Lin, Chen‐Jui Ho, Chih‐Hsiang Lin, Ya‐Yuan Hou, Cheng‐Han Chan, Meng‐Han Tsai

**Affiliations:** ^1^ Department of Neurology Kaohsiung Chang Gung Memorial Hospital Kaohsiung Taiwan; ^2^ Division of Cardiology, Department of Internal Medicine Taipei Veterans General Hospital Taipei Taiwan; ^3^ Department of Medical Research Kaohsiung Chang Gung Memorial Hospital Kaohsiung Taiwan; ^4^ School of Medicine Chang Gung University Taoyuan Taiwan

**Keywords:** ANK2, ANKB, arrhythmia, epilepsy, neurodevelopmental disorder

## Abstract

The Ankyrin 2 (*ANK2*) gene encodes the ankyrin‐B protein (ANKB), which is involved in the organization and stability of membrane ion channels, transporters, and receptors in cardiomyocytes and neurons. Variants in *ANK2* genes are initially reported in long QT syndrome and autism. Animal models with *ANK2* deletion have exhibited seizures and been anecdotally associated with epilepsy in case reports. Hereby, we reported a Taiwanese family with the *ANK2* pathogenic variant (chr4:114276707, c.6933del, p.T2312Lfs*2) that affects the giant ankyrin‐B isoform. The family members presented with young‐onset self‐limited focal epilepsy, and achieved seizure‐free in adulthood with antiseizure medications. Interestingly, the electrocardiogram revealed no obvious cardiac phenotype. We further reviewed reported *ANK2‐related* epilepsies. Most variants are de novo and loss‐of‐function variants. Most patients had young epilepsy or neonatal seizures. Notably, most cases of *ANK2*‐related epilepsy are self‐limited and pharmaco‐responsive, which suggests that it is likely to be underdiagnosed. With the increased availability of whole exome sequencing, the diagnosis of *ANK2*‐related epilepsies may increase. The co‐existence of QT prolongation on electrocardiogram, autism, and a positive family history of cardiac arrhythmia or sudden death may provide important clues in the clinical diagnosis of *ANK2*‐related epilepsy. Furthermore, a correct genetic diagnosis of *ANK2‐*related epilepsy will initiate close cardiac surveillance to avoid the potential sudden death risk of this disorder.

**Plain Language Summary:**

*ANK2* has long been regarded as an arrhythmic gene. This study reported the first familial *ANK2*‐related epilepsy, highlighting the role of *ANK2* in epileptogenesis. Most reported *ANK2*‐related epilepsies are self‐limited and pharmaco‐responsive, suggesting that they are likely to be underdiagnosed. Literature review of the phenotype and genotype of *ANK2* showed that LOF *ANK2* variants tend to have CNS phenotypes, whereas missense variants are arrhythmic. Early detection of *ANK2* variants in epilepsy patients is worthwhile considering the potential sudden death risk of this disorder.

**Key points**

- ANK2 pathogenic variants can cause epilepsies in addition to cardiac arrhythmia. Most ANK2 related epilepsies are self‐limited and pharmacoresponsive.
- ANK2 related epilepsies are likely underdiagnosed.
- Loss of function ANK2 variants tend to result in CNS phenotypes whereas missense variants are arrythmic.

## 1 INTRODUCTION

The Ankyrin 2 (*ANK2*) gene encodes the ankyrin‐B protein (ANKB), which belongs to the ANK family of proteins that serve as connectors between membrane proteins and the underlying cytoskeleton, contributing to cell shape, stability, and organization.^1^ ANKB, in particular, is involved in the organization and stability of membrane ion channels, transporters, and receptors in various cell types, including cardiac muscle cells and neurons,^2^ therefore affecting membrane excitability, and synaptic formation during development.

ANKB has several isoforms. The large 440 kDa isoform, giant ANKB (gANKB) is mainly expressed in neurons.^3^, ^4^ It contains a rod‐shaped neuron‐specific domain (NSD), which is formed by the inclusion of a large exon 38 between the spectrin binding domain (SBD) and the C‐terminal domain. gANKB is expressed in the neonatal brain,^5^ and decreases with age. The short 220‐kDa ANKB isoform is ubiquitously expressed in the heart and neurons postnatally.

Clinically, *ANK2* variants are initially associated with various cardiac conduction disorders^6^ highlighting its essential role in electrical signaling. Later, it is also associated with autism spectrum disorders (ASD). Variants in *ANK2* have been anecdotally associated with epilepsy, although the seizure semiology and epilepsy syndromes have not been well characterized.^7^, ^8^, ^9^ Given that ANKB is also highly expressed in neurons and involved in maintaining the organization of ion channels and receptors in the synapses, it is surprising that *ANK2*‐related epilepsies were reported scarcely.^10^ We herein report on a dominant inherited *ANK2* family presented predominantly with young‐onset self‐limited focal epilepsy and review relevant literature to further characterize this potentially under‐recognized condition.

## 2 METHODS

### 2.1 Whole exome sequencing (WES) study of the Taiwanese family

We identified a Taiwanese family in the epilepsy clinic of Kaohsiung Chang Gung Memorial Hospital with young‐onset focal epilepsy. The epilepsy was classified according to the International League Against Epilepsy report (ILAE).^11^ WES of the proband was performed through Epi25 collaboration (https://epi‐25.org/).^12^ In brief, the sample was captured using Illumina TruSeq Rapid Exome enrichment kit or TwistBioscience Human Core Exome kit and sequenced by HiSeq X or NovaSeq 6000 platform, followed by calling using the Genome Analysis Toolkit or Illumina DRAGEN pipeline, respectively. The variant calling files were analyzed using the Geneyx software (http://geneyx.com) and five variants remained (Table S2) after filtering for the gnomAD database and the severity was medium or severe (calculated by variant effect and multiple prediction algorithms). Only one variant existed in both the proband and her affected brother. This study was approved by the local Human Subject Research Ethics Committee, and written informed consents were obtained.

### 2.2 Literature review of patients with *ANK2* ‐related epilepsies

We searched the ClinVar database^13^ (Accessed on Feb. 1, 2024) and found 43 *ANK2* “pathogenic” or “likely pathogenic” variants. Large‐scale deletions or copy number changes were excluded because they may affect multiple genes. Variants without a provided condition or phenotype were also excluded, resulting in 14 variants. Additional 18 “pathogenic or likely pathogenic” variants from the UniProt database (https://www.uniprot.org)^14^ were also included. The associated phenotypes were categorized as follows: (I) cardiovascular (CV) phenotype, which included long QT syndrome and arrhythmias; (II) Neurodevelopmental disorders (NDD), which included ASD, intellectual disability (ID), and developmental delay (DD); (III) seizure or epilepsy; and (IV) miscellaneous, such as hyperammonemia, lactic acidosis or hypoglycemia…etc.

We also conducted a retrospective search of PubMed for patients with epilepsy caused by *ANK2* variants that may not be included in the ClinVar or Uniprot databases. Only cases with electroencephalography (EEG) descriptions and detailed information that fit the ILAE definition of epilepsy were included.^15^ To avoid confusion with convulsive syncope, cases presented with loss of consciousness with convulsions and concurrent arrhythmia on electrocardiography (ECG) without evidence of epileptiform discharges on EEG were excluded.

## 3 RESULTS

### 3.1 Novel *ANK2* pathogenic variant in a Taiwanese family (Figure 1)

**FIGURE 1:**
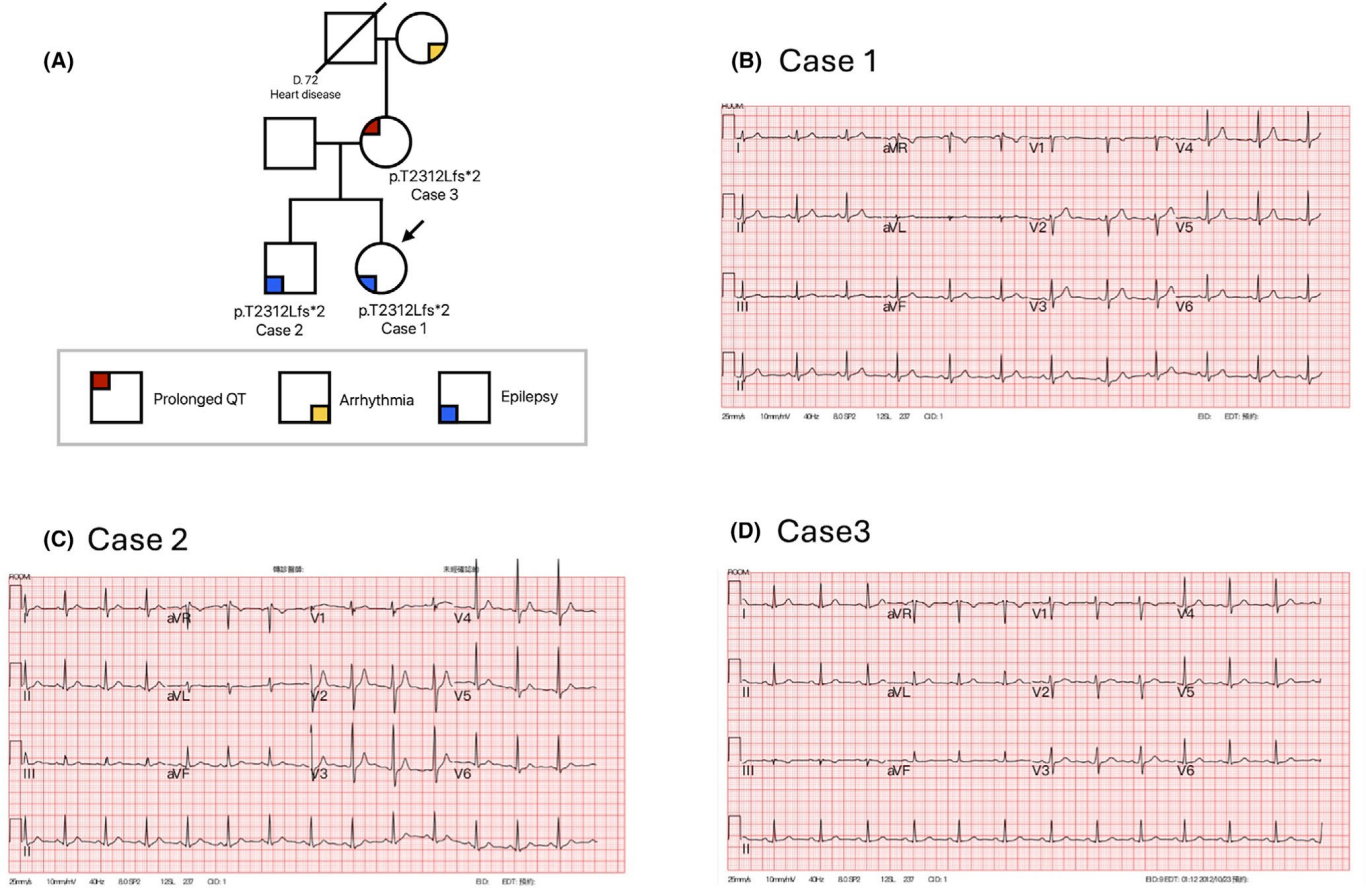
The pedigree and electrocardiography of the family with ANK2 frameshift variant. (A) The three cases detailed here carry the ANK2 pathogenic variant. The mother of the siblings had QT prolongation. The maternal grandmother, currently aged 93, also had arrhythmia. (B) Electrocardiogram from the index patient who suffered from ANK2‐related epilepsy. It showed a relatively broad T wave. (C) Traces from the sibling of ANK2‐related epilepsy. It showed a U wave and high amplitude T wave, and a late T wave over V6. (D) Traces from the asymptomatic individual who is the mother of case 1. It disclosed borderline QT prolongation (QTc = 461 ms) and notched T over V2.

The index patient (Case 1) came to our outpatient department for epilepsy and genetic consultation. The WES technique revealed that both the proband (Case 1) and her brother (Case 2) carried the same *ANK2* pathogenic variant.

#### 3.1.1 Case 1

This patient is a 24‐year‐old woman who had her first seizure at the age of six (Figure 1A). The aura manifested as ictal headache and dizziness, followed by bilateral tonic–clonic seizures. Focal epileptiform activity over the left frontocentral area with evolution to the bilateral hemisphere was documented on an EEG at 8 years old. Valproic acid was used before reaching her teenage years but later transitioned to lamotrigine due to considerations for potential future pregnancy. No episodes of seizure were reported after 14 years of age. Despite no clinical seizures, her EEG occasionally displayed abnormal epileptiform discharges. The brain magnetic resonance imaging (MRI) revealed a nonspecific subcortical hyperintense change on FLAIR in the bilateral parietal region. Resting ECG showed the corrected QT interval was 421 ms (normal range: <460 ms) (Figure 1B). The WES revealed a frameshift mutation resulting in premature truncation of the *ANK2* gene (c.6933del, p.T2312Lfs*2). The truncating variant located in the large exon 38, affects the neuron‐specific gANKB isoform, while the 220KDa isoform remains intact.

#### 3.1.2 Case 2

This 28‐year‐old male is the elder brother of Case 1 (Figure 1A). The patient had an episode of febrile seizures at 6 years old, and afebrile epilepsy from 7 years old. Frequent seizures were noted in his teenage years and ceased after the age of 18. The seizures tend to occur after exercise or missed antiseizure medication (ASMs). The seizures were controlled with valproate acid. He never had syncope. Sleep EEG recordings revealed a focal spike over the left frontal area at 10 years old. Although no clinical seizure noted after adulthood, interictal epileptiform discharges were still presented over the right frontal area (F4‐C4) at the age of 24. MRI of the brain was normal. His ECG showed a normal QT interval (Figure 1C). Sanger sequencing revealed the same *ANK2* pathogenic variant as Case 1.

#### 3.1.3 Case 3

This is the mother of Cases 1 and 2 (Figure 1A) and reported being in good health apart from a history of hypertension. She never experienced a seizure or syncope episode. Her ECG showed borderline QT prolongation (QTc = 461 ms) and notched T over V2 (Figure 1D). Sanger sequencing revealed that she carried the same *ANK2* pathogenic variant as her descendants. Her mother, now aged 93 had a history of arrhythmias but further medical recordings and blood were unavailable.

### 3.2 *ANK2* ‐related phenotypic spectrum

**FIGURE 2:**
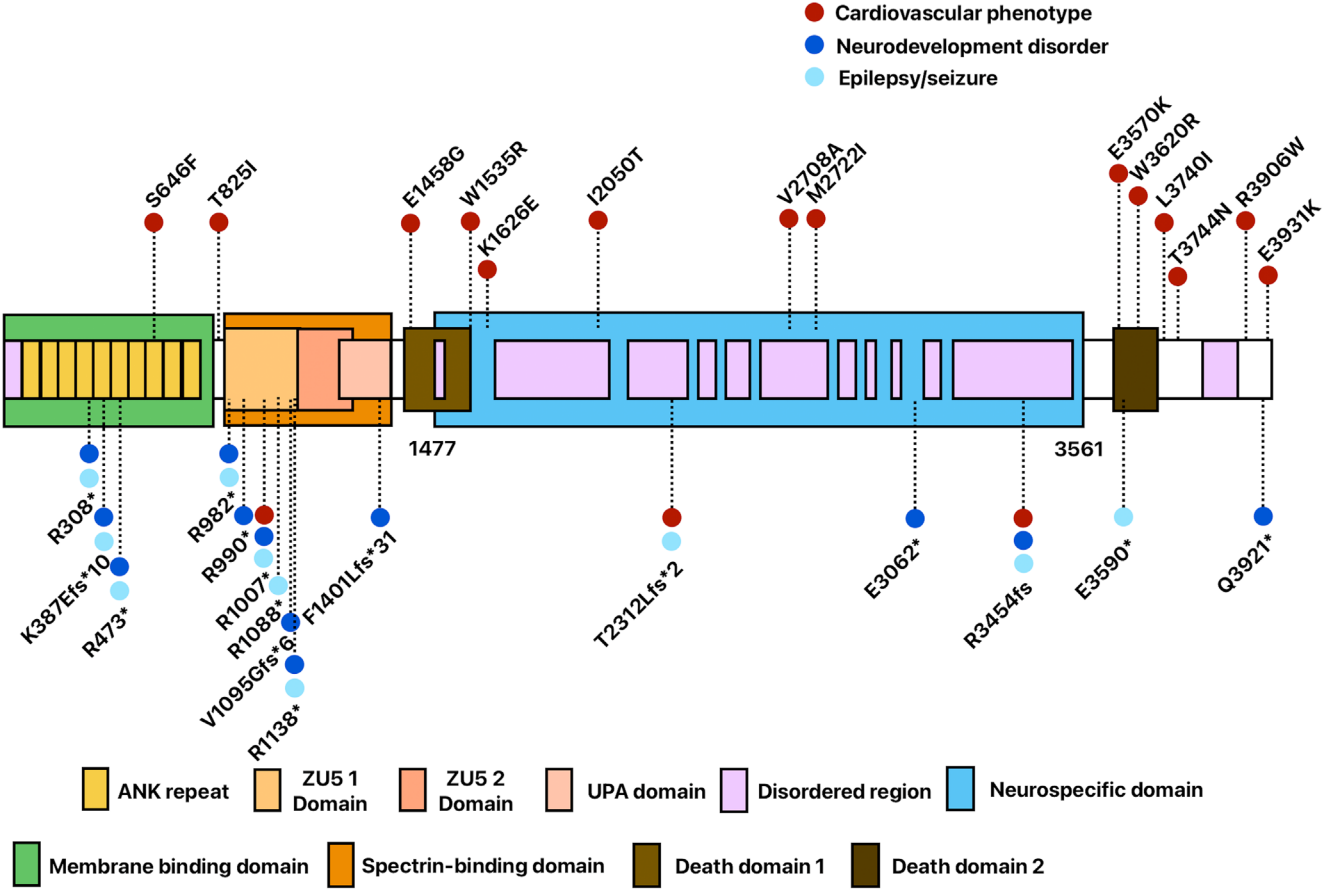
The distribution and type of human ANKB pathogenic/likely pathogenic variants. The predicted intrinsically disordered neurospecific domain region falls within 1477–3561 aa. Fifteen missense variants are drawn in the upper part. Fourteen loss‐of‐function variants are drawn in the lower part. CV phenotype is marked as a red dot. NDD is marked as a navy blue dot. Epilepsy or seizure is marked as a sky‐blue dot (Reference: UniProtKB Q01484‐4, 3957 aa).

A total of 32 pathogenic/likely pathogenic *ANK2* variants are reviewed from literature. The distribution of 29 pathogenic/likely pathogenic variants, consisting of 14 missense and 15 loss‐of‐function (LOF) variants was depicted on the canonical human ANKB (Figure 2). The remaining three were splice site variants (c.2179‐1G>A; c.2797‐1G>A; c.12881G>A), which were not shown in Figure 2. Two variants (c.2179‐1G>A; c.2797‐1G>A) are associated with NDD and epilepsy, and one (c.12881G>A) with epilepsy. Interestingly, all (14/14) missense variants are associated with the CV phenotype. Meanwhile, all (18/18) of the LOF variants are associated with CNS phenotype (NDD or epilepsy). Three (p.R1007*, p.T2312Lfs*2, p.R3454fs) among the 18 LOF variants are also associated with CV phenotype. There are 3 LOF variants located in the neuronal‐specific domain (NSD) between aa 1477–3561^18^ (p.T2312Lfs*, p.E3062*, p.R3454fs), all presented with NDD or seizures (Table S1).

### 3.3 *ANK2‐*related epilepsies

We identified 10 patients (including two reported here) with *ANK2*‐related epilepsy in the literature. Details of the clinical characteristics and genetic variants are listed in Table 1. All patients had LOF (nonsense, frameshift, or splicing) variants. Unlike previously reported cases of *ANK2*‐related epilepsy, which typically involved de novo mutations, our family appears to be dominantly inherited.

**TABLE 1:** Clinical and genetic information of patients with *ANK2*‐related epilepsy.

	Patient 1	Patient 2	Patient 3	Patient 4	Patient 5	Patient 6	Patient 7	Patient 8	Patient 9	Patient 10
cDNA location	c.6933del	c.6933del	c.2797‐1G>A	c.922C>T	c.2179‐1G>A	c.3019C>T	c.1159_1160del	C.12881G>A	c.10768G>T	c.1417C>T
Protein change	T2312Lfs*2	T2312Lfs*2	p.(?); –	R308*	p.(?); –	R1007*	K387Efs*10	p.(?); –	E3590*	R1138*
Mutation type	Frameshift	Frameshift	Splice site	Nonsense	Splice site	Nonsense	Frameshift	Splice site	Nonsense	Nonsense
Inheritance	Maternally inherited	Maternally inherited	De novo	De novo	De novo	De novo	De novo	De novo	De novo	De novo
Gender	Male	Female	Female	Male	Male	Male	Female	Male	Female	Female
ILAE classification	Focal epilepsy	Focal epilepsy	Neonatal seizures	Neonatal seizures	Focal epilepsy	West syndrome with infantile spasms and Lennox–Gastaut syndrome	Generalized epilepsy/epilepsy with myoclonic absences	Focal epilepsy	Focal epilepsy	Focal epilepsy
Age of onset	6 y/o	6 y/o	8 days	5 days	2 y/o	4 m/o	22 m/o	2 weeks	1 week	2 m/o
Precipitating factors	Drug discontinuation, fever	Drug discontinuation, hyperventilation, photic stimulation	n/a	n/a	n/a	n/a	n/a	n/a	n/a	n/a
Seizure timing	Mostly daylight	Mostly daylight	n/a	n/a	n/a	n/a	n/a	n/a	n/a	Either sleep or awake
EEG	Focal epileptiform activity over the right frontal area with evolution to left frontal area	Generalized rhythmic sharply contour theta activity and focal central rhythmic sharply contour delta activity	Multifocal epileptic discharges	Normal	Epileptiform discharges at right hemisphere	Multifocal epileptiform activity; left > right slowing	Generalized 3‐Hz spike and wave, progressive during sleep	Multifocal, mainly central (‐parietal) spikes	Focal central spikes	Normal background with interictal and ictal epileptiform discharges restricted to the midline central and right central regions
MRI	Subcortical hyperintensity on FLAIR in bilateral parietal region	Normal	Normal	Normal	n/a	Normal	Hyperintensities and atrophy	Normal	Normal	Normal
Treatment	VPA	VPA, and shifting to LTG considering pregnancy	LTG (history: VPA)	No	VPA 500/500 mg, clonidine 0.1 mg	Ketogenic diet, CZP, RFM, CLB, cannabinol, VPA, VNS	LTG, VNS (previous ASMs: VPA, CLB, ESM, TPM, LEV)	OXC	OXC	LEV, shifting to PB
Treatment response	Seizure‐free	Seizure‐free	Seizure‐free	Seizure‐free	Good to VPA	Resistant	Resistant	Seizure‐free	Seizure‐free	Partial response to LEV reached seizure‐free by PB
ID/DD	No	No	Mild	Mild	Mild	Severe	Moderate	No	No	Yes
ASD	No	No	Yes	Probable	Unknown	Yes	No	n/a	n/a	Yes
Cardiac problem	No	No	n/a	n/a	n/a	n/a	n/a	n/a	n/a	n/a
Reference	Our patients	Our patients	Teunissen et al.^9^	Teunissen et al.^9^	Teunissen et al.^9^	Teunissen et al.^9^	Teunissen et al.^9^	Teunissen et al.^9^	Teunissen et al.^9^	Teunissen et al.^9^

Abbreviations: ASD, autism spectrum disorder; ASM, antiseizure medications; CLB, clobazam; CZP, clonazepam; DD, developmental delay; EEG, electroencephalogram; FLAIR, fluid attenuated inversion recovery; ID, intellectual disability; LEV, levetiracetam; LTG, lamotrigine; MRI, magnetic resonance imaging; n/a, not available; OXC, oxcarbazepine; PB, phenobarbital; RFM, rufinamide; VNS, vagus nerve stimulation; VPA, valproic acid.

Most (8/10) *ANK2*‐related epilepsy manifest as young‐onset (aged from 2 months old to 6 years old) self‐limited focal epilepsy. Two were refractory to ASMs, including one West syndrome that evolved into Lennox Gastaut syndrome (Patient 6) and one epilepsy with myoclonic absences (Patient 7). Most (6/10, 60%) *ANK2* variants have ID or DD and the remaining had normal intellect. Of six patients with ID, only two have moderate to severe ID, and both have poor treatment responses.

In terms of treatment, one patient (Patient 4) was free from seizure without treatment. Sodium channel blockers, including lamotrigine, oxcarbazepine, or rufinamide, were the most commonly prescribed medications. Four patients (Patients 2, 3, 8, and 9) achieved seizure freedom with sodium channel blocker monotherapy. Phenobarbital and valproic acid were also utilized. Two patients (Patients 1 and 2) achieved seizure freedom with valproic acid monotherapy and Patient 10 achieved with phenobarbital only. The remaining three patients were refractory to multiple ASMs, among them, two patients (Patients 6 and 7) underwent vagus nerve stimulation implantation.

## 4 DISCUSSION

In this study, we reported a Taiwanese family carrying a novel *ANK2* pathogenic variant (chr4:114276707, c.6933del, p.T2312Lfs*2) which specifically affects the neuron‐specific gANKB isoform. A literature review of *ANK2*‐related epilepsy demonstrates that the majority of patients (8 out of 10) have young‐onset (<6 years old) self‐limited focal epilepsy or neonatal seizure, which mostly respond well to ASM monotherapy. Although epilepsy was not initially identified in *ANK2*‐related disorders, it can be the main presentation in some cases, like our family. Due to its self‐limited and pharmaco‐responsive nature, it may be underdiagnosed.

The role of *ANK2* in epileptogenesis was supported by animal studies, mice with prenatal *ANK2* deletion leads to remodeled proteosome in the cortical synapse, causing seizures, hyperactivity, and social deficit.^16^ A study of heterozygous LOF *ANK2* human‐induced pluripotent stem cells demonstrated reduced ANKB expression leads to hyperactive and desynchronized neuronal network activity.^9^ It is suggested that *ANK2* variants disrupt proper neuronal ion channel function, thereby affecting their excitability and contributing to seizure activity.^9^

ANKB is known to interact with multiple ion channels and transporters. It interacts with Cav2.1, and Cav2.2 channels in the central nervous system; whereas in the heart system, it interacts with IP3R, Cav1.3, NCX, Na^+^/K^+^ ATPase and SERCA2.^10^ The absence of *ANK2* can reduce the expression of the sodium/calcium exchanger and L‐type, T‐type, and P/Q‐type voltage‐gated calcium channels in both cardiomyocytes and neurons.^2^ The aforementioned voltage‐gated channels, situated at the presynaptic terminals, permit the influx of calcium ions, thereby regulating the release of neurotransmitters and/or hormones. Since both the brain and heart are vital organs that rely on electrophysiological activities to function properly, abnormal calcium homeostasis resulting in aberrant synaptic transmission is likely underlying the disease mechanism.^16^ It remains unclear why LOF and missense *ANK2* variants are presented with different phenotypes. It is possible that LOF variants affecting short ANKB are mostly embryonic fatal when expressed in cardiac tissue. Alternatively, it is also possible that ANKB affects different channels/transporters in the brain and heart, thus different consequences. More studies may be required to investigate the observation.

Epilepsy‐related *ANK2* variants are all LOF variants, including nonsense, frameshift, and splicing variants while the pathogenic missense variants are all associated with CV phenotype. But this distinction is not absolute, LOF variants can still be linked with CV phenotype. The majority of previously reported *ANK2* variants were de novo. Here, we reported the first dominantly inherited epilepsy family. Epilepsy‐related variants can influence both isoforms of ANKB, however, variants located in the neuron‐specific giant exon appear to have a milder phenotype. This may be due to the short 220 kDa adult isoform not being subject to nonsense‐mediated decay if the variants are located in the NSD domain of the giant neuronal isoform. This may also explain why the seizures in our siblings resolved with age because gANKB expresses more abundantly at the early age of development and decreases with age.

Interestingly, most long QT syndrome‐related variants are missense, affecting both the short ANKB and gANKB isoforms containing the NSD domain. Although gANKB is predominantly expressed in the brain, it is also expressed in the heart tissue to a lesser amount (according to GTEx Portal, https://www.gtexportal.org/home/). It is also possible that ANKB can be expressed in the heart conducting system, which utilizes calcium signaling to regulate cardiac rhythm. Missense variants may translate into dysfunctional gANKB proteins in the cardiac conduction system that causing QT prolongation.

Clinically, convulsive syncope and epilepsy share common presentations.^17^ The key to differentiating these two conditions relies on video‐EEG.^17^ This issue is particularly more bothersome in *ANK2*‐related disorders because they can have both cardiac and neurological abnormalities. Our family, however, demonstrated that *ANK2* pathogenic variants can be associated with familial focal epilepsies without CV phenotypes. The prevalence of *ANK2* pathogenic variants in apparent self‐limited focal epilepsies may be underrated and worth further investigation.

In terms of treatment, sodium channel blockers and broad‐spectrum ASM valproate acid, appear to be effective in the treatment of *ANK2*‐related epilepsy.^8^, ^9^ Interestingly, perampanel (an α‐amino‐3‐hydroxy‐5‐methyl‐4‐isoxazolepropionic acid receptor antagonist) normalizes neuronal network activity and improves survival in the *ANK2* deletion mice model.^16^ This suggests that circuit hyperactivity may be caused, at least in part, by increased AMPA receptor‐mediated synaptic transmission. The effect of perampanel on *ANK2‐*related seizures in humans remains to be determined.

The limitation of this study is the retrospective nature of the literature review and the small number of reported *ANK2*‐related epilepsy. Given the mild epilepsy phenotype without obvious ASD and cardiac manifestations, we believed *ANK2*‐related epilepsies may be under‐recognized and underdiagnosed. The increasing access to NGS technology may unveil more *ANK2* cases in the future and provide a more comprehensive picture of *ANK2*‐related disorders. Finally, sudden death has been reported in *ANK2* families with predominant CV phenotypes.^6^ It remains unclear whether the risk of sudden death is also increased in patients with predominant brain phenotypes. Patients with *ANK2*‐related ASD and epilepsies may benefit from intensive cardiac monitoring and preventive measures if prompt genetic diagnosis can be achieved.

## AUTHOR CONTRIBUTIONS

P‐HL conducted the literature review, summarized the clinical phenotyping, and interviewed the family. M‐HT conducted the clinical phenotyping, initiated this study, and motivated the collaborators. C‐HL gave valuable advice on the frame of the scripts. Y‐YH handled statistical issues. C‐HC reviewed and interpreted the family's ECGs. P‐HL, and M‐HT analyzed and interpreted the data. P‐HL, C‐HL, C‐JH, Y‐YH, C‐HC, and M‐HT drafted the manuscript. All authors read and revised the manuscript.

## FUNDING INFORMATION

This study is supported by the National Science and Technology Council (113‐2314‐B‐182‐060‐MY3), National Health Research Institute (NHRI NHRI‐EX112‐11022NI), and Chang Gung Memorial Hospital (CORPG8P0141).

## CONFLICT OF INTEREST STATEMENT

None of the authors has any conflict of interest to disclose.

## ETHICS STATEMENT

We confirm that we have read the Journal's position on issues involved in ethical publication and affirm that this report is consistent with those guidelines.

## Supporting information


Table S1.

Table S2.
