# Supplementary material for: Self‐limited familial focal epilepsy caused by ANK2 variants: A potentially under‐recognized condition
Source: Epilepsia Open. 2025 Feb 17;10(2):635–42. doi: 10.1002/epi4.70003 (PMC12014929; doi:10.1002/epi4.70003)
Supplement: Supplementary file 1 — Table S1. [file EPI4-10-635-s001.docx]

**Supplement Table1. *ANK2* variants in ClinVar**

| **Nucleotide HGVS** | **Protein HGVS** | **Mutation type** | **Clinical presentation** | **Clinical significance** | **Publication** |
| --- | --- | --- | --- | --- | --- |
| c.1417C>T | p.R473* | Nonsense | Aggressive behavior, behavioral abnormality, delayed speech and language development, epileptic spasm, global DD, hyperammonemia, infantile spasms, ID, microcephaly, seizure | Likely pathogenic | Stevens and Rasband 2022 |
| c.2944C>T | p.R982* | Nonsense | ¶CV phenotype, seizures, language impairment, speech articulation difficulties, DD, abnormality of the optic nerve, delayed speech and language development, short stature, chronic diarrhea, dolichocephaly, deeply set eye, frontal bossing, generalized hypotonia, small for gestational age, recurrent hypoglycemia, episodic fever, lactic acidosis, chronic neutropenia, motor aphasia, recurrent bacterial skin infections | Pathogenic |  |
| c.3019C>T | p.R1007* | Nonsense | Cardiac arrhythmia, ankyrin-B-related | Likely pathogenic |  |
| c.3262C>T | p.R1088* | Nonsense | Epileptic encephalopathy, seizure | Pathogenic | Swayne, Murphy et al. 2017 |
| c.3281dup | p.V1095Gfs*6 | Frameshift | ¶CV phenotype, NDD | Pathogenic |  |
| c.4203del | p.F1401Lfs*31 | Frameshift | §Complex NDD | Likely pathogenic |  |
| c.4876A>G | p.K1626E | Missense | Long QT syndrome | Likely pathogenic | De Rubeis, He et al. 2014 |
| c.6149T>C | p.I2050T | Missense | Long QT syndrome | Likely pathogenic | De Rubeis, He et al. 2014 |
| c.8123T>C | p.V2708A | Missense | Long QT syndrome | Likely pathogenic | De Rubeis, He et al. 2014 |
| c.9184G>T | p.E3062* | Nonsense | ASD | Pathogenic |  |
| c.10360del | p.R3454fs | Frameshift | Autistic disorder of childhood onset, growth hormone deficiency, DD, seizures, aortic dilatation, chest pain, migraine, paresthesia, failure to thrive, delayed skeletal maturation, tics, ADHD, sleep disturbance, mood changes, dental crowding, pectus excavatum, hyperextensibility at elbow, clumsiness, malar flattening, upslanted palpebral fissure, macrotia, abnormality of the lip, heart murmur, asthma, muscle weakness, bruising susceptibility, visual impairment | Likely pathogenic | Cunha and Mohler 2009 |
| c.11760_11761delinsTT | p.Q3921* | Nonsense | ¶CV phenotype, NDD | Pathogenic | Shigemizu, Aiba et al. 2015 |
| c.11761C>T | p.Q3921* | Nonsense | ¶CV phenotype, autistic disorder of childhood onset, developmental regression, decreased body weight, esotropia, long fingers, hyperactivity | Pathogenic | Shigemizu, Aiba et al. 2015 |
| c.2179-1G>A |  | Splice acceptor | Seizures, mild ID, global DD, ADHD, developmental regression, pain insensitivity, EMG abnormality, peripheral neuropathy, aggressive behavior, tall stature | Likely pathogenic |  |

Reference: NM_001148.6

§This variant is classified as likely pathogenic based on ACMG/AMP guidelines for variant interpretation, but other variants that introduce a premature termination codon in this region and downstream have been detected in individuals and are considered pathogenic.

¶This variant is labeled as CV phenotype in ClinVar, but it is expected to be causative of *ANK2*-related NDD. The evidence for the gene-disease relationship is limited for cardiac disease.

Abbreviation: ADHD=attention deficit hyperactivity disorder; CV=cardiovascular; DD=developmental delay; EMG=electromyography; ID=intellectual disability; NDD=neurodevelopmental disorder
