# Supplementary material for: Self‐limited familial focal epilepsy caused by ANK2 variants: A potentially under‐recognized condition
Source: Epilepsia Open. 2025 Feb 17;10(2):635–42. doi: 10.1002/epi4.70003 (PMC12014929; doi:10.1002/epi4.70003)
Supplement: Supplementary file 2 — Table S2. [file EPI4-10-635-s002.docx]

**Supplement Table2. Candidate Variants identified in the family**

|  | | **Genomic and genetic data** | | | | **ACMG classification** | **Clinical evidence** | | **Effect & prediction** | |
| --- | --- | --- | --- | --- | --- | --- | --- | --- | --- | --- |
| Location | Gene | REF | ALT | AA | ZYG |  | OMIM | OMIM Inheritance | Effect | Severity |
| 4:114276705 | ANK2 | GC | G | T2312Lfs*2 | HET | LP | Cardiac arrhythmia, ankyrin-B-related; Long QT syndrome type 4 | AD | Frameshift | High |
| 18:31325883 | ASXL3 | CT | C | P2026Lfs*54 | HET | VUS | Brainbridge-Ropers syndrome | AD | Frameshift | High |
| 1:155161537 | MUC1 | T | G | D752A | HOM | VUS | Tubulointerstitial kidney disease, autosomal dominant, 2 | AD | Missense | Medium |
| 11:31811460 | PAX6 | CT | CTT | E396Gfs*7 | HET | Benign | Coloboma of optic nerve; Morning glory disc anomaly; Aniridia; Anterior segment dysgenesis 5 | AD | Frameshift | High |
| 12:50479223 | SMARCD1 | C | T | A24V | HOM | VUS | Coffin-Siris syndrome 11 | AD | Missense | Medium |

Abbreviations: AA=amino acid; AD=autosomal dominant; ALT=alternative; HET=heterozygous; HOM=homozygous; LP=likely pathogenic; REF=reference; VUS=variant of uncertain significance; ZYG=zygosity
